# Defective maturation of dendritic cells during HIV-1 infection is associated with increased expression of SOCS-1

**DOI:** 10.1186/1471-2334-12-S1-P88

**Published:** 2012-05-04

**Authors:** Meenakshi Sachdeva, Aman Sharma, Sunil K Arora

**Affiliations:** 1 Post Graduate Institute of Medical Education and Research (PGIMER), Chandigarh, India

## Background

During chronic HIV-1 infection, upregulation in the expression of certain negative regulatory factors has been implicated recently as a cause of defects in dendritic cells (DCs). We aim to study the association of one such factor, the suppressor of cytokine signaling-1 (SOCS-1) gene with DC dysfunction during HIV-1 infection.

## Methods

DCs from 21 therapy naïve (mean CD4: 256 cells/mm^3^), 21 patients on anti-retroviral therapy (mean CD4: 342 cells/mm^3^) and 14 healthy controls were immunophenotyped for maturation markers at baseline and after 5 hour *ex vivo* stimulation with TLR-4 ligand, LPS, by flowcytometry. Subsequently, the expression of SOCS-1 gene and the cytokine levels were assessed in monocyte-derived DCs (Mo-DC) of healthy donors exposed to LPS and HIV-1 gp120 by real time PCR and flowcytometry respectively.

## Results

The myeloid DCs of untreated subjects had significantly lower responsiveness to LPS stimulation as indicated by lower upregulation of CD83 (mean±SE: 31±4.4 vs. 50±3) and CD80 (30±4 vs. 40±3) as compared to healthy controls. Treated patients had a higher upregulation of CD83 (mean±SE: 38±4) and CD80 (mean±SE: 33±3) though not significantly higher than untreated patients. The expression of SOCS-1 was higher upon exposure to HIV-1 gp120 than LPS in 5 healthy controls assessed and their culture supernatants showed decreased levels of all the cytokines, mainly IL-6 and TNF-α.

## Conclusions

Therapy naïve patients exhibit deficient DC maturation upon LPS stimulation, which is partially restored following antiretroviral treatment. An increased expression of SOCS-1 gene upon gp120 exposure suggests a possible role of SOCS-1 in DC impairment.

